# The Effect of Computerized Cognitive Behavioral Therapy on People's Anxiety and Depression During the 6 Months of Wuhan's Lockdown of COVID-19 Epidemic: A Pilot Study

**DOI:** 10.3389/fpsyg.2021.687165

**Published:** 2021-07-14

**Authors:** Zhangwei Lv, Jinyang Li, Bin Zhang, Ning Zhang, Chun Wang

**Affiliations:** ^1^Nanjing Brain Hospital, Nanjing Medical University, Nanjing, China; ^2^Cognitive Behavioral Therapy Institute of Nanjing Medical University, Nanjing, China; ^3^Department of Psychiatry, Nanfang Hospital, Southern Medical University, Guangzhou, China

**Keywords:** COVID-19, anxiety, depression, lockdown of Wuhan, computerized cognitive behavioral therapy

## Abstract

**Background:** The effectiveness of computerized cognitive behavioral therapy (CCBT) has been proven for mild and moderate anxiety and depression. In 2016, the first official Chinese CCBT system was launched by Chinese Cognitive Behavior Therapy Professional Organizations and included four items: getting out of depression, overcoming anxiety, staying away from insomnia and facing Obsessive-compulsive disorder. During the COVID-19 epidemic, Chinese CCBT system served the public for free. This study explored the effects of CCBT on anxiety and depression by comparing the use of the platform during the epidemic and during the same period in 2019.

**Methods:** Users were divided into a depression group or an anxiety group according to their own discretion. The subjects used the self-rating anxiety scale (SAS) and self-rating depression scale (SDS) before each training. Each training group completed the corresponding CCBT training project, which had 5–6 training sessions, an average of once every 5 days. The training content in 2019 and 2020 was identical. This study compared the demographic characteristics, depression, and anxiety levels of CCBT platform users during the lockdown period in Wuhan (LP2020), where the outbreak was concentrated in China, from January 23 to July 23, 2020 and the same period in 2019 (SP2019).

**Result:** (1) There were significant differences in gender (χ*2* = 7.215, *P* = 0.007), region (χ*2* = 4.225, *P* = 0.040) and duration of illness (χ*2* = 7.867, *P* = 0.049) between the two periods. (2) There was a positive Pearson correlation between the number of users of CCBT platform during LP2020 and number of confirmed cases of COVID-19 in each province (*r* = 0.9429, *P* < 0.001). (3) In LP2020, the SAS (*t* = 2.579, *P* = 0.011) and SDS (*t* = 2.894, *P* = 0.004) scores at T0 in Hubei were significantly higher than those in other regions. (4) The CCBT platform has an obvious effect on anxiety (*F* = 4.74, *P* = 0.009) and depression on users (*F* = 4.44, *P* = 0.009).

**Conclusion:** This study showed women, students and people who are more seriously affected by the epidemic were more likely to accept the CCBT training. The CCBT platform made a significant contribution toward alleviating the anxiety and depression symptoms of users during the epidemic. When face-to-face psychotherapy is not available during the epidemic, CCBT can be used as an effective alternative.

## Introduction

At the end of 2019, the epidemic caused by COVID-19 (SARS-CoV-2) suddenly hit, causing a serious impact on the politics, economy and society in various countries around the world (Lai et al., 2020). In order to prevent and control the COVID-19 epidemic and effectively cut off the spread of the virus, the Chinese government enacted city lockdown measures on Wuhan beginning 10:00 on January 23, 2020. The country's emergency measures successfully delayed the spread of the epidemic and ultimately limited the scale of COVID-19 (Tian et al., 2020).

Measures such as isolation and lockdown achieved the expected results, however, they resulted in unintended consequences for people's mental health. For instance, the social and physical distancing measures of quarantine turned out to be key risk factors for mental health issues. A multinational study showed that starting from March 2020, 19.1% of Chinese respondents were at risk of severe mental illness (95% CI: 16.9–21.6%), and 16.6% of British respondents were at risk of severe mental illness (95% CI: 14.6–18.8%) (Goodwin et al., 2021). Furthermore, the investigation of a non-clinical, non-infected sample showed that in the early stage of the COVID-19 epidemic, the anxiety level of respondents increased significantly, and the anxiety level of women increased more than that of men (De Pietri and Chiorri, 2021). In addition, a study assessed 1,036 children and adolescents quarantined as a result of the COVID-19 epidemic, of which 112 (11.78%) cases of depression and 196 (18.92%) cases of anxiety were identified; 68 (6.56%) cases presented both (Chen et al., 2020).

A recent study has shown that the COVID-19 epidemic will have a serious impact on the mental health of many people, but the negative psychological consequences can be minimized by taking corresponding intervention measures (Paredes et al., 2021). Although many forms of psychotherapy are effective in treating depression, cognitive behavioral therapy (CBT) is by far the most well-studied form of psychotherapy for depression (Cuijpers, 2017). A total of 115 unique studies identified from 127 publications were eventually included in a meta-Analysis. In summary, the sample included 7,719 patients and the final results consistently proved the effectiveness of CBT in reducing anxiety symptoms in adolescents (Wang et al., 2017), which is consistent with the results of a previous systematic review (Manassis et al., 2010). Besides, other studies have provided robust evidence for the effectiveness of CBT in the treatment of anxiety and depressive disorders in adolescents and young adults compared with passive controls (A-Tjak et al., 2021; Riise et al., 2021; Wakefield et al., 2021; Wergeland et al., 2021). These studies indicate that CBT is a great choice for alleviating the mental health problems caused by COVID-19.

Due to the extreme infectiousness of COVID-19 and the isolation and lockdown measures taken to prevent the epidemic, classical treatment methods such as traditional face-to-face interactive psychological evaluation and intervention have been hindered (Alqahtani et al., 2021). With the therapist and patient being isolated, innovative approaches need to be taken in order to continue providing excellent medical services while minimizing the risk of exposure to or spreading of COVID-19. The obstacles impeding face-to-face psychotherapy can be solved by computerized cognitive behavioral therapy. For patients, CCBT can reduce barriers to access mental health resources in remote areas (Anderson et al., 2004), especially since it is so urgently needed by patients during the epidemic. Another advantage of CCBT is that it can be used to enhance follow-up treatment. CCBT can also reduce the shame of requiring psychiatric treatment (Carlbring et al., 2011). Further, trained therapists can increase their ability to provide mental services by using CCBT (Andrews and Erskine, 2002; Anderson et al., 2004). Additionally, because all forms of CCBT use less therapeutic support than conventional psychotherapy, CCBT has been demonstrated as a cost-effective strategy (Eells et al., 2014; Andersson et al., 2015; Wright et al., 2019; Wright and Caudill, 2020). Because of its effectiveness, acceptability and feasibility, CCBT has been popular since its inception in the 1990s (Andersson et al., 2019) and its effectiveness for treating mild and moderate depression and anxiety has been proven by many studies since (Bowler et al., 2012; Carlbring et al., 2018; Kuechler et al., 2019; Wright et al., 2019; Christ et al., 2020). Thus, computerized cognitive behavioral therapy is the most suitable form of psychotherapy in isolation due to its convenience, non-contact, and effectiveness.

In April 2016, Chinese Cognitive Behavioral Therapy Professional Organization launched China's first official CCBT system and its effectiveness against depression and anxiety has since been verified by numerous studies (Li et al., 2018). In order to mitigate the effects of the epidemic, CCBT was made available to the public for free during the COVID-19 epidemic. The purpose of this study is to explore the level of public anxiety and depression during the epidemic and which people tended to choose CCBT platform, and to explore the effect of CCBT platform on relieving public anxiety and depression.

## Methods

### Materials

The CCBT platform (http://CCBT.cbtchina.com.cn and mobile phone “CCBT” APP) has been online since April 15, 2016. The CCBT platform includes four training projects: getting out of depression, overcoming anxiety, staying away from insomnia and facing obsessive-compulsive disorder. Users who did not select anxiety or depression programs among those registered in SP2019 and LP2020 were screened out. Users' general information including age, gender, ethnicity, geographic area and region, occupation and clinical variables (e.g., other psychological problems, duration of illness, onset frequency, medical visits, physical disease, etc.) were collected. All outcome measures were collected digitally via the same digital platform where patients accessed the training.

### Participants

**Figure 1:**
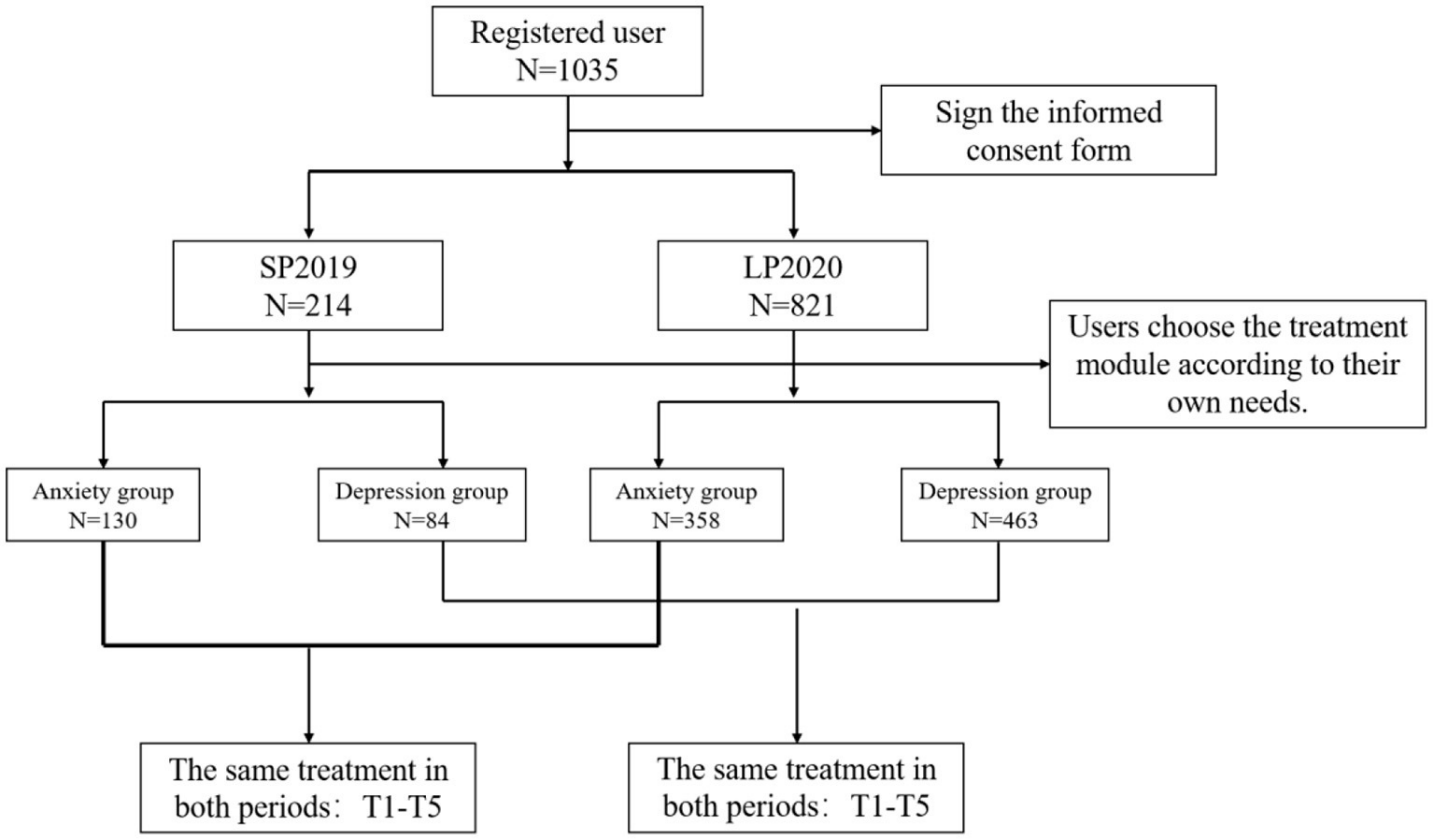
Diagram of participant flow.

Users registered on the CCBT platform by searching on the Internet or in the mobile application platform and selected a training project according to their own needs. When registering for the CCBT platform, all users participate voluntarily and sign informed consent. All users who registered during SP2019 and LP2020 and used anxiety and depression programs in CCBT platform were included in this study. A user who did not complete the training or did not log in again for more than 90 days was defined as a dropout. In SP2019 and LP2020, 214 and 821 users signed up for the CCBT platform, respectively. Their ages ranged from 10 to 73. There were 712 (68.79%) women. Figure 1 is a diagram of flow of participants through the study. This study was reviewed and approved by the Ethics Committee of Nanjing Medical University.

### Self-Rating Measures

In the anxiety group, symptoms were assessed using the self-rating anxiety scale (SAS) (Zung, 1971). SAS measures 20 items across a total of six training sessions and adopts a four-level scoring method with scores ranging from 1 to 4. A higher score indicates more severe symptoms. According to Chinese norm results, a total score from 50 to 59 is classified as mild anxiety, from 60 to 69 as moderate anxiety, and above 70 as severe anxiety. In the depression group, the self-rating depression scale (SDS) (Zung, 1965) was used to evaluate symptoms. The SDS evaluates 20 items across six training sessions and adopts a four-level scoring method with scores ranging from 1 to 4. A higher score indicates more severe symptoms. Users conducted self-assessment of symptoms before each training. According to Chinese norm results, a total score of 53–62 is classified as mild depression, 63–72 as moderate depression, and more than 72 as severe depression. We recorded the level of anxiety or depression before the first training as the baseline level (T0), and asked users to complete a symptom assessment before each subsequent training (T1–T5).

### Intervention Measures

The depression group and the anxiety group completed their corresponding CCBT training projects, respectively. Each project was set with six training sessions, which were conducted once every 5 days on average. The training sessions of each project were the same in both time periods. The contents of each training in the anxiety group and depression group are shown in the Table 1.

**Table 1:** Theme of six training sessions for users in the anxiety group and depression group.

	**Anxiety group**	**Depression group**
Session 1	Anxiety and CBT model	Depression and CBT model
Session 2	Automatic thought restructuring and relaxation training	Automatic thought restructuring and behavior activation
Session 3	Distorted cognition and anxiety rating list	Distorted cognition and function behavior
Session 4	Exposure training and breathing training	Change in attribution Style and task Decomposition
Session 5	Core belief and positive orientation	Core belief and problem-solving techniques
Session 6	Review of goals and plans, frustration response, and recurrence prevention	Review of goals and plans, frustration response, and recurrence prevention

### Data Analysis

The chi-square test was used to compare the differences in demographic characteristics, depression and anxiety levels between the two time periods. Correlation analysis was used to compare the relationship between the number of confirmed cases of COVID-19 announced by the Chinese government (HNC, 2021) in different provinces and the number of CCBT users. One-way repeated measure analysis of variance (ANOVA) was used to analyze the trend of anxiety and depression symptoms after each training. Repeated measure ANOVA was used to analyze multiple scores on the symptom scales in the anxiety and depression groups. Due to the very high dropout rate in SP2019, we regard the effect after three trainings as the indicator of therapeutic effect. The Kolmogorov-Smirnov Test was used to complete the normal test of all count data, and the homogeneity test of variance was carried out for the normal distribution data. All data analysis was performed in SPSS 21.0 statistical software (IBM, Chicago, IL, USA), and data is presented in the form of average and standard deviation (*M* ± *SD*), or number and percentage. GraphPad Prism 6.02 (GraphPad Software Inc., San Diego, California, USA) was utilized for plotting of graphs. Statistical significance was set by *p*-values of < 0.05.

## Results

### Comparison of Demographic Characteristics Among Users of CCBT Platform in SP2019 and LP2020

The chi-square test was used to compare the demographic characteristics of users in the two periods (Table 2). The results showed that there were significant differences in gender (χ*2* = 7.215, *P* = 0.007), region (χ*2* = 4.225, *P* = 0.040) and duration of illness (χ*2* = 7.867, *P* = 0.049) between the two periods. Among all users, most were aged 19–27 (34.36%) and 28–36 (34.46%). Out of 1,035 participants, 712 were female, the mean age was 29.01 years (range = 10–73; *SD* = 10.01) and the dropout rate was 27.50%. The proportion of students was 13.43%. For most users, the incidence of disease was within the past 3 months (81.12%), it was the first onset (86.21%) and no other physical diseases were reported (85.89%).

**Table 2:** Comparison of demographic characteristics of CCBT platform users in the two periods.

	**SP2019 (*N* = 214)**	**LP2020 (*N* = 821)**	***χ2***	***P***
**Age group, y^a^**				
10–18	12	50	8.267	0.142
19–27	75	261		
28–36	73	264		
37–45	18	131		
46–54	12	62		
Over 54	4	16		
**Gender**				
Male	83	240	7.215	**0.007**
Female	131	581		
**Occupation**				
Student	29	110	0.003	0.953
Other	185	711		
**Region^b^**				
Han nationality	175	782	4.225	**0.040**
Minority	13	29		
**Duration of illness^c^**				
Within 3 months	154	628	7.867	**0.049**
3 months−1 year	8	51		
1–5 years	13	53		
Over 5 years	19	38		
**Onset frequency^d^**				
1 time	105	664	0.021	0.989
2–5 times	9	58		
Over 5 times	8	48		
**Physical disease**				
Yes	38	108	2.968	0.085
No	176	713		

a *Missing value: n = 57(5.51%);*

b *missing value: n = 36(3.48%);*

c *missing value: n = 71(6.86%);*

d *missing value: n = 143(13.82%)*.

*The meaning of bold words indicates P-value < 0.05*.

### Differences in Demographic Characteristics and Number of Users of CCBT Platform in Different Regions During the Epidemic

**Figure 2:**
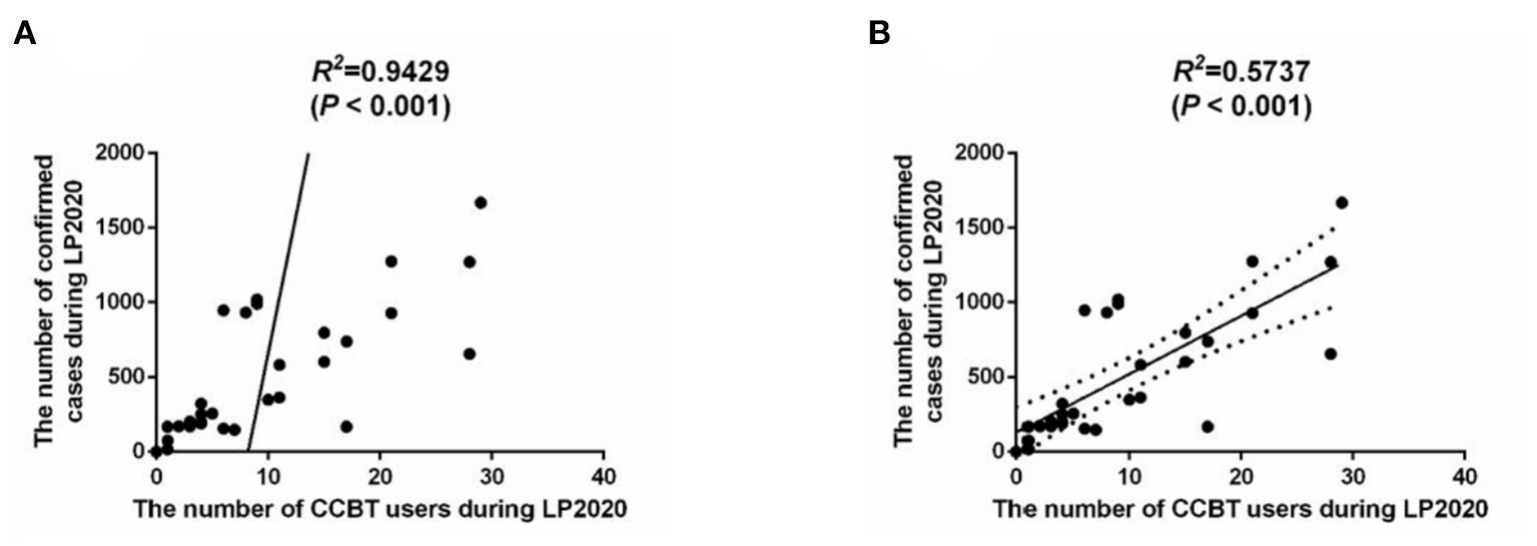
**(A)** Correlation analysis of the number of people confirmed cases of COVID-19 and the number of users of the CCBT platform during LP2020. **(B)** Correlation analysis of the number of people confirmed cases of COVID-19 and the number of users of the CCBT platform during LP2020 without Hubei province.

In LP2020, there were significant differences between CCBT users from Hubei province and those not from Hubei province in terms of occupational distribution (χ*2* = 25.534, *P* = 0.001), region distribution (χ*2* = 5.172, *P* = 0.023) and treatment situation (χ*2* = 6.855, *P* = 0.009). Regardless of province, students made up the highest proportion of users. The proportion of minorities among users from Hubei (3.80%) was lower than that of users who were not from Hubei (5.12%). Users in Hubei displayed a greater tendency to seek help at professional medical institutions (23.75%). Pearson correlation between the number of users of CCBT platform during LP2020 and number of confirmed cases of COVID-19 in each province was positive (*r* = 0.9710, *P* < 0.001) (Figure 2A). During LP2020, the number of CCBT users in Hubei Province was 181 (37.63%), and the number of confirmed cases was 68,135 (81.36%). After removing the extreme value of Hubei Province, there was a positive correlation between the number of users and the number of confirmed cases (*r* = 0.7574, *P* < 0.0001) (Figure 2B).

### Dropout Rate

The dropout rates in SP2019 and LP2020 were 94.71 and 10.92%, respectively. Further analysis showed that in LP2020, there were significant differences in the dropout rates at different ages (χ*2* = 7.572, *P* = 0.006) with medical visits (χ*2* = 6.481, *P* = 0.011) and physical diseases (χ*2* = 7.572, *P* = 0.006). The dropout rate of users aged 37–45 (8.87%) without medical visits (4.90%) or physical diseases (1.96%) was lower.

### Anxiety and Depression in CCBT Users

**Figure 3:**
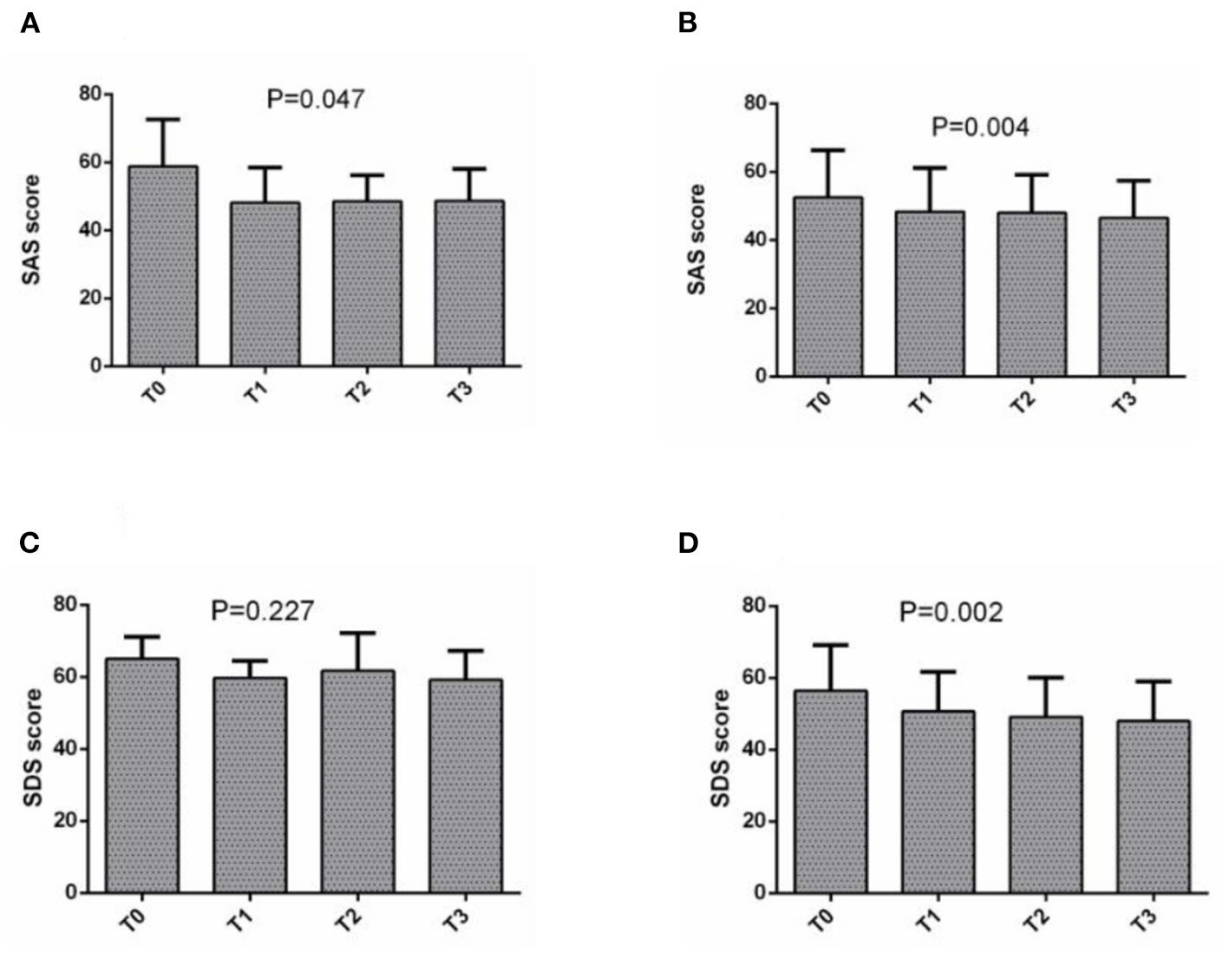
**(A)** The change of SAS score in SP2019, **(B)** the change of SAS score in LP2020, **(C)** the change of SDS score in SP2019, and **(D)** the change of SAS score in LP2020.

In the anxiety group, training had the effect of gradually decreasing SAS score in LP2020 (*P* = 0.047) and SP2019 (*P* = 0.004). In the depression group, the SDS score (*P* = 0.002) also continued to decline (Figure 3). An unpaired *t*-test was used to examine anxiety and depression symptoms among CCBT users before training in SP2019 and LP2020. The results showed that there was no significant difference in users' anxiety symptoms between the two periods, but depression levels in SP2019 were higher than in LP2020 (*t* = 6.751, *P* < 0.001). For LP2020, SAS (*t* = 2.579, *P* = 0.011) and SDS (*t* = 2.894, *P* = 0.004) scores at T0 in Hubei were significantly higher than in other regions.

### Effect of CCBT on Anxiety and Depression

**Figure 4:**
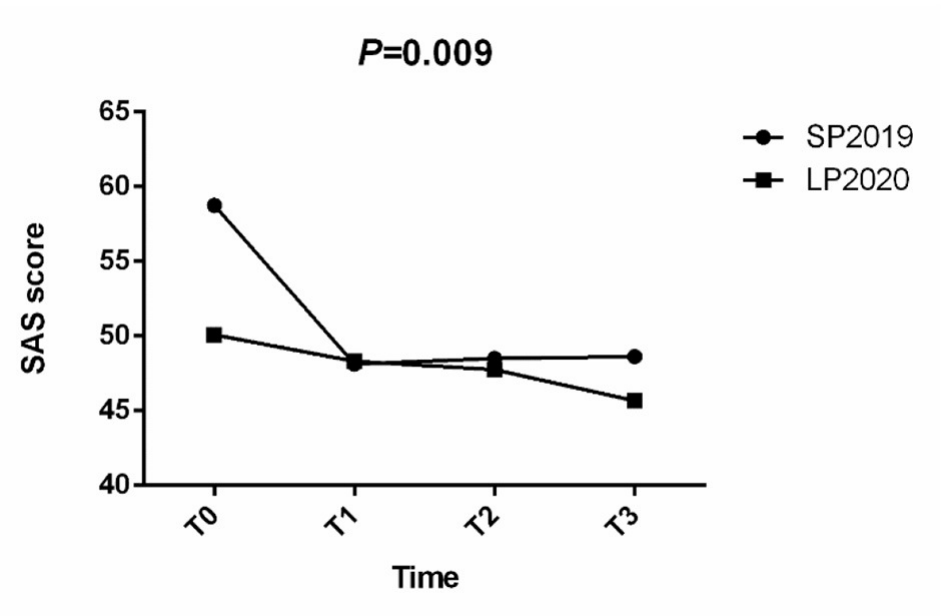
SAS scores at T0, T1, T2, and T3, by group (SP2019, LP2020).

**Figure 5:**
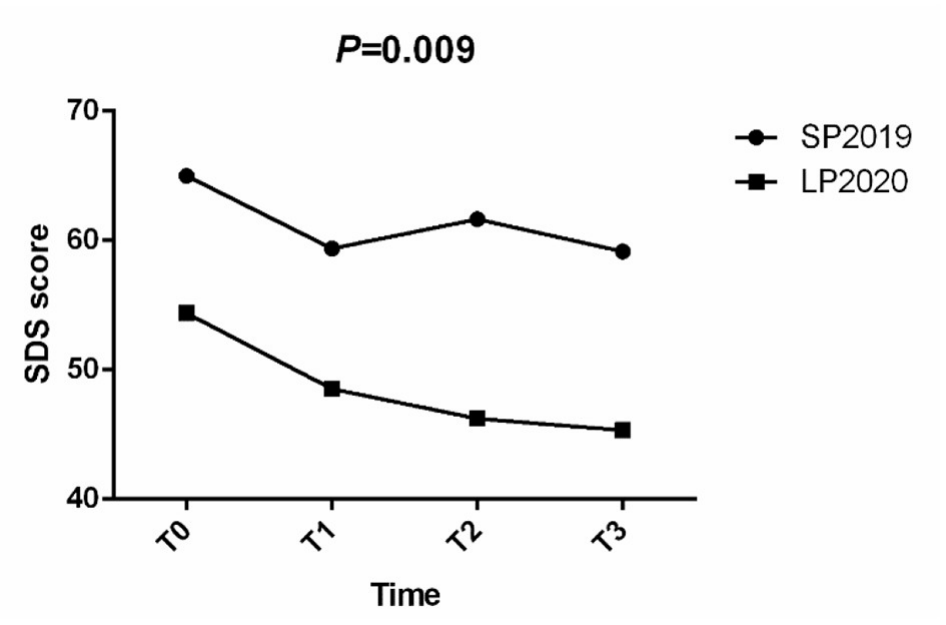
SDS scores at T0, T1, T2, and T3, by group (SP2019, LP2020).

A repeated measures ANOVA was conducted to compare the mean scores on the SAS and SDS before and after CCBT training. Group (SP2019 vs. LP2020) was included as the between-subjects factor. The results showed that in the anxiety group, the interaction between time period and training times was not significant (Figure 4), but the main effect of training times was significant (*F* = 4.742, *P* = 0.009). In the depression group, the main effect of training times was significant (*F* = 4.438, *P* = 0.009), but the interaction between training times and time period was not significant (Figure 5).

## Discussion

In the present study, CCBT was used as a substitute for traditional face-to-face CBT to help people affected by the epidemic. CCBT was shown to be effective for anxiety and depression during lockdown. This study provides important findings about the mental health status and the likelihood of people of different geographical locations, ages and genders to use CCBT, which can help decision makers design targeted interventions and allocate resources reasonably in order to effectively improve specific mental health status.

Compared with other regions, CCBT users in Hubei Province (an epicenter of the epidemic in China) displayed more severe anxiety and depression. This finding is in agreement with a previous study suggesting that during the rising stage of the outbreak, psychological and behavioral responses of the masses are significant (Zhang et al., 2020). Based on the relationship between the confirmed number of cases and the number of CCBT users, it can be extrapolated that as the severity of the epidemic grows, the number of residents using CCBT will increase. In particular, younger users, women and people from Hubei are more likely to use the CCBT platform, which is consistent with the results of previous studies (Li et al., 2018).

In this study, we found that people between the ages of 19 and 27 had higher levels of anxiety and depression and were more likely to use CCBT. In contrast, anxiety level before training in SP2019 and LP2020 decreased with age. This finding is consistent with the results of similar studies (Balsamo and Carlucci, 2020). Early studies have also shown that young people are more likely to have mental health problems after an outbreak of a disease (Main et al., 2011). This may be because compared with other age groups, the youth group (19–27) receives more distressing information on the Internet and exhibits reduced psychological resilience (Cheng et al., 2014), prompting them to choose CCBT platform to alleviate their anxiety and depression levels. In terms of gender differences, in LP2020, women accounted for a larger proportion of CCBT users, which may be caused by a higher degree of psychological distress among women than men (Ho et al., 2020). This evidence suggests that women are more sensitive to stressors than men (Wang et al., 2020), and that women engage in self-help behaviors as a result. In terms of geographical differences, anxiety and depression levels in Hubei Province, the center of China's epidemic, were higher than those not from Hubei in LP2020, but there was no significant difference in distribution between the two regions in SP2019. The residents in the areas most affected by the epidemic experienced higher levels of anxiety and depression, which is consistent with the results of previous studies (Balsamo and Carlucci, 2020). A cross-sectional study found that anxiety and depressive symptoms were positively associated with current residence in Wuhan and college locations based in Wuhan (Wu et al., 2021). Another study showed that location is very influential on mental health, with residents in big cities displaying higher levels of anxiety and depression than participants living in rural areas (Zhong et al., 2020). During the epidemic, residential area was an important predictor of anxiety and depression (Lenzo et al., 2020). For example, residents in Hubei Province isolated at home, so inadequate access to daily necessities and medical care may explain increased anxiety and depressive symptoms compared to other provinces. In the absence of face-to-face medical treatment, looking for computerized treatment on the Internet has become the best choice, especially during the epidemic. CCBT not only saves medical resources, it also helps to reduce inequalities in access to health care (Jaffe et al., 2020).

In this study, the dropout rates of CCBT in SP2019 and LP2020 are 94.71 and 10.92%, respectively. Previous studies have also pointed out that CCBT has a higher dropout rate than traditional face-to-face psychotherapy (Cai et al., 2015). Due to the lack of doctor's guidance, this computer-based, self-service, unsupervised treatment method not only shows its efficiency and convenience, but also reduces the compliance of users (Melville et al., 2011). In view of this situation, some researchers believe that on the basis of the standardized guiding theoretical model, the matching degree between CCBT and its users should be improved from demographic variables such as patients' gender, age, education level and stressful life events; on the other hand, the setting and content of CCBT should be improved to enhance its attractiveness to patients (Zhang and Qian, 2018).

CCBT users reduced their SAS and SDS scores through training in SP2019 and LP2020, indicating that CCBT is effective in relieving anxiety and depression. This is consistent with results of a previous meta-analysis (Andrews et al., 2018). Because CCBT is effective and acceptable for patients with anxiety and depression (Ebert et al., 2015), some researchers recommend that CCBT may be a promising treatment option when face-to-face treatment is not feasible (Wright et al., 2019; De Luca and Calabrò, 2020). Because CCBT reduces medical costs while ensuring positive training effects, CCBT can be used not only as a regular tool for patients with anxiety and depression, but also as a resource in times of emergency.

In this study, we discovered that there was a correlation between the number of confirmed cases of COVID-19 and the number of CCBT users. To be more specific, there were more CCBT users in provinces with a more severe epidemic. This should inform policy makers that the allocation of health care should be skewed toward areas with severe outbreaks, and that the use of CCBT is recommended.

Based on the results of this study, psychological intervention needs to be implemented during the epidemic, especially in areas where the epidemic is more serious. Our study found that public anxiety and depression levels increased in areas with severe outbreaks, so more medical resources should be allocated to these areas.

There are some limitations in this study. First of all, participants were recruited through the Internet, and there is no guarantee that users will complete all treatment. This resulted in a high dropout rate which affected the evaluation of the effectiveness of CCBT. Secondly, in this study, only the self-rating anxiety and depression scale were used to evaluate the symptoms of the users, so the symptoms could not be evaluated comprehensively or accurately. Third, users' attitudes, experience and feedback on the CCBT platform were not collected. Although the number of users in LP2020 was much higher than in SP2019, the reason for the increase cannot be accurately explained. Fourth, in order to explore the effectiveness of the CCBT platform in the actual application scenario, this study did not set up a randomized controlled trial, which will be added to follow-up studies to explore how CCBT works.

## Conclusion

This study showed that young CCBT users from Hubei Province had higher levels of anxiety and depression during the first 6 months of the lockdown of Wuhan. Women, students and people who are more seriously affected by the epidemic were more likely to accept the CCBT training. The CCBT platform made a significant contribution toward alleviating the anxiety and depression symptoms of users during the epidemic. When face-to-face psychotherapy is not available during the epidemic, CCBT can be used as an effective alternative.

## Data Availability Statement

The original contributions presented in the study are included in the article/supplementary material, further inquiries can be directed to the corresponding author.

## Ethics Statement

The studies involving human participants were reviewed and approved by the Ethics Committee of the affiliated Nanjing Brain Hospital of Nanjing Medical University. Written informed consent to participate in this study was provided by the participants' legal guardian/next of kin.

## Author Contributions

BZ designed and built the CCBT platform. JL completed the data collection and sorting. ZL completed the data analysis and manuscript writing. After reviewing the manuscript, CW and NZ proposed amendments. All authors approved the submitted version.

## Conflict of Interest

The authors declare that the research was conducted in the absence of any commercial or financial relationships that could be construed as a potential conflict of interest.
